# Accredited Medical Web: an experience in Spain

**DOI:** 10.2196/jmir.2.suppl2.e5

**Published:** 2000-09-13

**Authors:** R Sarrias, MA Mayer, M Latorre

**Affiliations:** ^1^Official Medical College of BarcelonaSpain

## Abstract

**Introduction:**

The Official Medical College of Barcelona (OMCB) is a centennial corporation created to defend the interests of the medical profession and ensure that it adheres to ethical and scientific norms in order to offer the best healthcare services to society. The Internet has the capacity to transmit a volume of information that is both difficult to control and widely available. The OMCB emphasizes the necessity to contribute to the accreditation of medical/healthcare information diffused through the network, and the importance of offering support and assistance, whenever necessary, to physicians and medical institutions who wish to access this information and be present within the Internet.

**Objective:**

The promotion from Official Medical College of Barcelona-Metges on line (OMCB) in Spain of an accredited medical web Seal (now called WMA of *METGES on line*) to give a reference guidelines of high quality based on Ethical Standards of the HON Code and the OMCB itself.

**Method:**

Through a registration form the person in charge of the website asks for the accreditation web seal by accepting the rules proposed (Code of Conduct, Accreditation, Identification, Confidentiality, Control and validation, Advertising and other sources of financing, Non-compliance and responsibilities) . Then a peer-review of experts assesses the content, the site ownership, date of posting, revising, and updating and other characteristics defined in the guidelines. Later, the peer-reviewer sends a mail to explain the acceptance or not acceptance and the improvement necessary to receive the web seal in a second review. This is a free process. The accredited website will carry the medical seal on the homepage.

**Results:**

At the moment there are 76 webs accredited (Biomedical Associations, Patient Associations, Medical Institutions, Training, Scientific Societies, NGOs of Development, Conferences). Only 30% were initially accepted before the modifications needed to get the web seal were made. The main reason for not acceptance are: the dates that content is posted, revised, and updated are not clearly indicated and the source for specific content is not clearly identified (ie, author, organizational, institutional, or commercial provider/producer). There are 30 websites waiting for accreditation and everyday the OCMB is receiving new requests.

**Conclusions:**

1. There is a big interest by all webs with medical contents to be accredited by a medical official institution as OMCB.
2. The differences of contents and guidance of the websites make it difficult to use only one set of criteria to accredit these webs and makes it necessary to modify our Code to be able to assess their heterogeneousness.
3. It would be necessary to have a system of different types of web seal to accredit the webs to guide professional and patient consumers and it would be very interesting to create an intelligent web data system to aid the peer-review the websites.

